# Three new species of *Hagnagora* Druce, 1885 (Lepidoptera, Geometridae, Larentiinae) from Ecuador and Costa Rica and a concise revision of the genus

**DOI:** 10.3897/zookeys.537.6090

**Published:** 2015-11-18

**Authors:** Gunnar Brehm

**Affiliations:** 1 Institut für Spezielle Zoologie und Evolutionsbiologie mit Phyletischem Museum, Vor dem Neutor 1, 07743 Jena, Germany

**Keywords:** Taxonomy, *Hagnagora*, Costa Rica, Ecuador

## Abstract

Three new *Hagnagora* Druce species (Geometridae, Larentiinae) are described: *Hagnagora richardi* Brehm, **sp. n.** from Ecuador, *Hagnagora hedwigae* Brehm, **sp. n.** from Ecuador, and *Hagnagora mirandahenrichae* Brehm, **sp. n.** from Costa Rica. A checklist of taxa assigned to *Hagnagora* is provided. *Hagnagora* is provisionally divided into six clades: the *anicata* clade (6 species), the *buckleyi* clade (3 species), the *croceitincta* clade (3 species), the *ephestris* clade (3 species), the *mortipax* clade (4 species) and *Hagnagora subrosea* (1 species). Two taxa are revived from synonymy: *Hagnagora catagrammina* Druce, **stat. rev.** and *Hagnagora luteoradiata* Thierry-Mieg, **stat. rev.** Two taxa are reinstated from subspecies to species level: *Hagnagora acothysta* Schaus, **stat. rev.** and *Hagnagora jamaicensis* Schaus, **stat. rev.** Four taxa are provisionally removed from *Hagnagora*: *“Hagnagora” ignipennis*, *“Hagnagora” mesenata*, *“Hagnagora” vittata*, and *“Hagnagora” ceraria*. After these changes, the genus *Hagnagora* now comprises 20 valid species.

## Introduction

The Neotropical genus *Hagnagora* was invented by Druce (1885a) and described by Druce (1885b). So far, it comprised 23 described taxa, with 16 valid species (Parsons et al. 1999, Brehm and Sullivan 2005, Sullivan 2011). One species, *Hagnagora mortipax* Butler, was subdivided into three subspecies. Eighty-three percent of all taxa had been described by 1913, followed by one taxon described in 1927 and three over the last decade. This pattern appears typical for Neotropical geometrid genera (Brehm et al. 2011). The assignment of taxa to *Hagnagora* is largely based on the Lepidoptera card index of the Natural History Museum in London (NHM), and subsequently from the catalogue of geometrid moths (Parsons et al. 1999). During identification work on Ecuadorian and Costa Rican geometrid moths, it became obvious that *Hagnagora* – like most Neotropical geometrid genera – requires revision. In this paper, I attempt to solve some of the most urgent taxonomic problems of the genus. I describe three new species, revive two species from synonymy, transfer two species from subspecies to species level, and provisionally exclude four taxa from the genus. With one exception, all known taxa assigned to *Hagnagora* are illustrated. I also include available molecular genetic data (COI gene) in order to aid species identification. This consice revision will be a basis of future taxonomic work that will be required, e.g. with regard to the question whether *Hagnagora* is monophyletic or consists of two separate lineages.

## Species identities and Barcode of Life Data Systems

Species delimitation and description of Lepidoptera has traditionally focused on their external morphology, mostly wing patterns. These formed the basis of all original descriptions of taxa assigned to *Hagnagora* in past centuries. The latest three species descriptions additionally contain not only colour plates, but also illustrations of male and female genitalia (Brehm and Sullivan 2005, Sullivan 2011). Additionally, molecular genetic information is available for these three taxa in the form of sequence data for the 658 bp fragment of the mitochondrial Cytochrome Oxidase I gene (“COI-barcodes”). A system of unique Barcode Index Numbers (BINs) has been established by Barcode of Life Data Systems (www.boldsystems.org) (Ratnasingham and Hebert 2013). It usually allows a quick and reliable assignment of specimens to other specimens in the system, whether identified to species or higher taxonomic level. Barcoding of all type specimens is an ultimate goal that would greatly increase the reliability of identifications, particularly in poorly studied tropical regions, and in cryptic and ‘difficult’ arthropod taxa. However, while barcoding of old type specimens is possible and relatively cheap (Strutzenberger et al. 2012), financial and bureaucratic constraints are still impeding a large-scale molecular analysis of type specimens in museums. In this paper, assignment of barcodes to described species was therefore performed by careful comparison of type material with freshly collected material, and all respective specimens are illustrated. The assignments are working hypotheses until original types are eventually barcoded. Twelve different BINs are assigned to different *Hagnagora* species in this paper, thus covering a substantial part of the known taxa. In one case, one BIN has been assigned to two taxa that nonetheless are treated as morphologically separate species.

To accelarate the taxonomic progress and following a recently reached consensus amongst geometrid taxonomists (Forum Herbulot 2014), this study focuses not on extensive species descriptions, but on diagnostic characters and the synthesis of illustrated external characters, genitalia structures and COI barcodes.

## Methods

Moths were pinned and dissected following established techniques (Lafontaine 2004, Hünefeld et al. 2011). Genitalia slides were embedded in Euparal, stained with Chlorazol Black, and digitised using an Olympus dotSlide system with 10x magnification. Adult moths were photographed in raw format using a 60 mm Nikkor macro lens mounted on a Nikon D700 camera. Photos were adjusted and colour plates were mounted using Photoshop and InDesign software (Adobe Systems, San José, USA).

**Figure 1.**
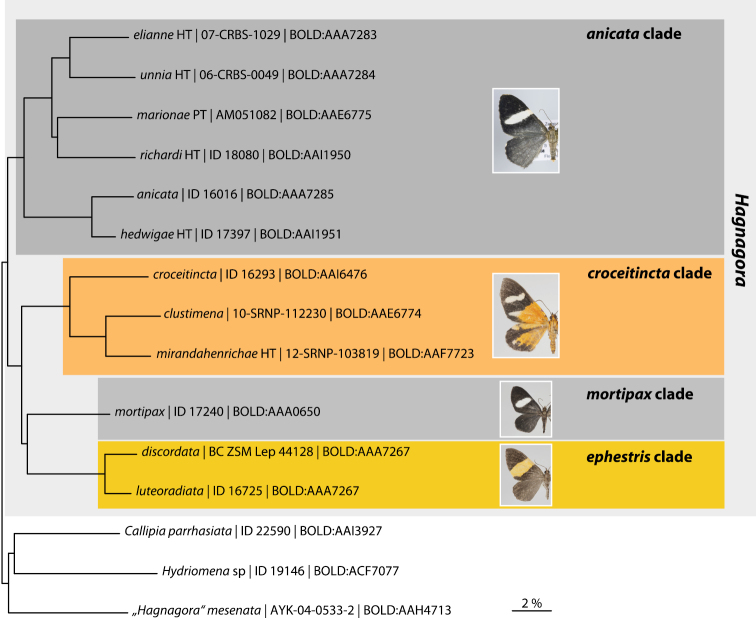
Summary tree of the available molecular genetic data based on genetic COI ‘barcodes’ using the Kimura 2 parameter implemented in BOLD systems. Four out of six clades are represented by the barcode data; no data were available for the *buckleyi* clade and for *Hagnagora subrosea*. *“Hagnagora” mesenata* groups outside *Hagnagora* sensu stricto. The species name is followed by the individual identification number and the Barcode Index Number (BIN). HT: Holotype, PT: Paratype.

Sequencing of the barcode fragment of the COI gene was carried out at the Canadian Center for DNA barcoding in Guelph, Ontario. Barcode sequences were compared by nearest neighbour analyses (Kimura 2 parameter), as implemented on the Barcode of Life Data Systems website (Ratnasingham and Hebert 2007). The resulting trees represent preliminary hypotheses of taxa groupings and can form the basis of future phylogenetic work. Fig. 1 shows a summary tree of all available taxa with barcode data. It visualizes similarities and differences in the COI gene between the different taxa and it was instrumental in differentiating four of the six provisional larger clades indentified within *Hagnagora*.

The following acronyms are used for institutions in which the specimens are deposited:

CISEC Colección de Invertebrados del Sur del Ecuador, Universidad Tecnica Particular Loja, Ecuador

NHM Natural History Museum, London, UK

PMJ Phyletisches Museum, Jena, Germany

RCGB Research Collection Gunnar Brehm, Jena, Germany

SMF Senckenberg Museum, Frankfurt a. M., Germany

SMNS Staatliches Museum für Naturkunde, Stuttgart, Germany

USNM National Museum of Natural History [formerly United States National Museum], Washington D.C., USA

ZSM Zoologische Staatssammlung, München, Germany

## Results and Discussion

### Distribution and Biology

Species previously assigned to *Hagnagora* were described from a wide range of Central and South American countries ranging from Mexico and Jamaica (17–18° N) to Chile (Valdivia province, ca. 39° S). Table 1 provides an overview of all taxa. The southernmost type locality of any *Hagnagora* species considered in this paper is Valparaíso in Chile (33° S) for *Hagnagora discordata*, but this record needs confirmation. Judging from their type localities, most species have a predominantly montane distribution. This includes the three recently described Costa Rican species (Brehm and Sullivan 2005, Sullivan 2011), as well as species described from the Colombian, Ecuadorian, Peruvian and Chilean Andes and mountains in SE Brazil.

**Table 1.** Overview of taxa assigned to *Hagnagora* and excluded from the genus, sorted according to six provisional clades, ordered alphabetically. LT Lectotype, HT Holotype, ST Syntypes.

Taxon	Author	Year	Described in	Country	Type Locality	BIN	Museum	Types
**1 *buckleyi* clade (3 sp)**								
*buckleyi*	Druce	1885	*Hagnagora*	Ecuador (north)	[Imbabura, Intag] Intaj	no	NHM	ST
*catagrammina* stat. rev.	Druce	1885	*Hagnagora*	Nicaragua, Panama	Nicaragua: Chontales; Panama: Volcán de Chiriqui; Bugaba, 800–1500 ft	no	NHM	ST
*lex*	Druce	1885	*Hagnagora*	Ecuador (east)	[Pastaza] Sarayacu	no	NHM	ST?
**2 *anicata* clade (6 sp)**								
*anicata*	Felder & Rogenhofer	1875	*Heterusia*	[Colombia]	Bogotá	assigned: BOLD:AAA7285	NHM	LT
*elianne*	Sullivan	2011	*Hagnagora*	Costa Rica	Alajuela Province: Volcan Poás, Alajuela Province	BOLD:AAA7283	USNM	HT
*hedwigae* sp. n.	Brehm	this paper	*Hagnagora*	Ecuador	Zamora-Chinchipe	BOLD:AAI1951	PMJ	HT
*marionae*	Brehm & Sullivan	2005	*Hagnagora*	Costa Rica	Heredia, Braulio Carrillo	BOLD:AAE6775	SMNS	HT
*richardi* sp. n.	Brehm	this paper	*Hagnagora*	Ecuador	Zamora-Chinchipe	BOLD:AAI1950	PMJ	HT
*unnia*	Sullivan	2011	*Hagnagora*	Costa Rica	Tapantí National Park, Cartago Province, 1275m; Volcan Poás, Alajuela Province, 2500m, Villa Mills, Cartago Province, 2841m	BOLD:AAA7284	USNM	HT
**3 *croceitincta* clade (3 sp)**								
*croceitincta*	Dognin	1892	*Polythrena*	[Ecador, (south)]	Loja (surroundings)	assigned: BOLD:AAI6476	USNM	HT
*epimena*	Bastelberger	1908	*Heterusia*	Peru (east)	Cuschi [Cushi]		SMF	ST
*clustimena*	Druce	1893	*Heterusia*	Mexico, Panama	Mexico: Coatepec; Panama: Chiriqui	assigned: BOLD:AAE6774	NHM	ST
*mirandahenrichae* sp. n.	Brehm	this paper		Costa Rica	Guanacaste	BOLD:AAF7723	PMJ	HT
**4 *mortipax* clade (4 sp)**								
*mortipax*	Butler	1872	*Scordylia*	Costa Rica	?	assigned: BOLD:AAA0650	NHM	ST?
*flavipectus*	Warren	1897	*Heterusia*	[Colombia]	Bogotá	(no)	NHM	HT
*jamaicensis* stat. rev.	Schaus	1901	*Heterusia*	Jamaica	?	no	USNM	ST?
*acothysta* stat. rev.	Schaus	1901	*Heterusia*	[Brazil]	Parana, Castro	no	USNM	ST?
*guatica*	Schaus	1927	*Scordylia*	Guatemala	Volcan Sta. Maria	no	USNM	ST?
**5 *ephestris* clade (3 sp)**								
*ephestris*	Felder & Rogenhofer	1875	*Heterusia*?	[Colombia]	Bogota	no	NHM	ST?
*discordata*	Guenée in Boisduval & Guenée	[1858]	*Scordylia*	[Chile]	Valparaíso	assigned: BOLD:AAA7267	NHM	ST
*luteoradiata* stat. rev.	Thierry-Mieg	1892	*Heterusia*	Costa Rica, Bolivia	?	assigned: BOLD:AAA7267	USNM	ST
**6 *subrosea***								
*subrosea*	Warren	1909	*Cophocerotis*	Peru (south east)	Carabaya, Oconeque, 7000 ft	no	NHM	ST?
**Species provisionally removed from the genus: *“Hagnagora”***								
*ignipennis*	Dognin	1913	*Heterusia*	Colombia	Bogotá, 2800–3200 m	no	USNM	ST
*mesenata*	Felder & Rogenhofer	1875	*Heterusia*	Chile	?	assigned: BOLD:AAH4713	NHM	ST
*vittata*	Philippi	1859	*Euclidia*	Chile	Provincia de Valdivia	380 bp fragment	?	ST
*ceraria*	Molina	1782	*Phalaena*	Chile	?	no	?	ST

Caterpillars of *Hagnagora* are only known for *Hagnagora mortipax* and *Hagnagora luteoradiata* from NW Costa Rica (Janzen and Hallwachs 2014; http://janzen.bio.upenn.edu/caterpillars/database.lasso). Both species’ caterpilars were recorded on *Clethra mexicana* DC. (Ericales, Clethraceae) (Figs 42, 43). One species excluded in this paper from *Hagnagora*, i.e. *“Hagnagora” vittata*, was reared in captivity on *Fuchsia magellanica* LAM. (Onagraceae) (King and Parra 2011). King and Parra (2011) also described the morphology of the egg and larva of *“Hagnagora” vittata*.

*Hagnagora* imagines mandatorily fold their wings vertically while resting in the same way as most butterflies (Fig. 41). They share this behaviour with genera such as *Callipia* Guenée and *Erateina* Doubleday (personal observations), whereas most geometrids display different resting positions. *Hagnagora* species are frequently observed at night and are readily attracted to artificial light sources (Brehm 2002, Brehm and Sullivan 2005), while both diurnal and nocturnal activity has been recorded for *Hagnagora marionae* and *“Hagnagora” vittata* (Brehm and Sullivan 2005, King and Parra 2011). Furthermore, both mud puddling and diurnal activity of *Hagnagora mortipax* has been observed in Peru (www.flickr.com/photos/76033499@N00/15919107346/). Apart from these isolated observations, little is known about the behaviour and ecology of *Hagnagora* moths.

### *Hagnagora*: a monophyletic genus?

**Figures 2–6.**
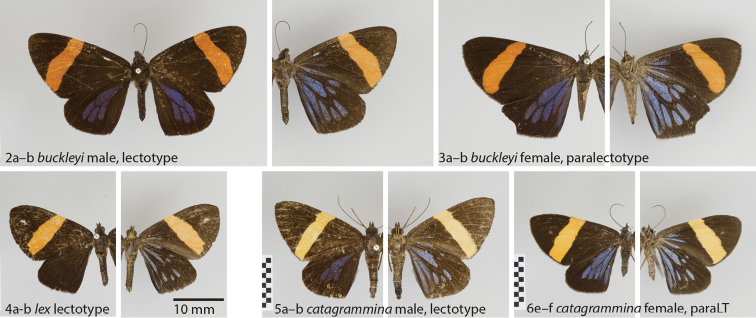
**2** *Hagnagora buckleyi* Druce male, lectotype **a** dorsal view **b** ventral view **3** *Hagnagora buckleyi* female, paralectotype **a** dorsal view **b** ventral view **4** *Hagnagora lex* Druce male, lectotype **a** dorsal view **b** ventral view **5** *Hagnagora catagrammina* Druce male, lectotype **a** dorsal view **b** ventral view **6** *Hagnagora catagrammina* Druce female, paralectotype (paraLT) **a** dorsal view **b** ventral view.

Druce (1885a) described *Hagnagora buckleyi* and *Hagnagora lex*, and shortly defined the genus together with the description of *Hagnagora catagrammina* (Druce 1885b). Druce (1885b) established *Hagnagora* largely by comparison with *Anemplocia splendens* (Druce, 1885) due to differences in the wing shape. The colourful *Hagnagora buckleyi* clade shares an apparently unique combination of wing pattern characters (Figs 2–6): The forewings have an orange transversal band, and the hindwings display fields of metallic blue between the veins. Notably, Druce (1893) did not include *Hagnagora clustimena* Druce, *Hagnagora discordata* Gn, and *Hagnagora mortipax* Butler in *Hagnagora*, but assigned them to *Heterusia* Hübner. Recent molecular genetic studies have shown that *Heterusia* sp. and *Hagnagora mortipax* are closely related, but do not form a monophyletic group (Sihvonen et al. 2011). Parsons et al. (1999), following the card index of the Natural History Museum, transferred several species previously assigned to *Heterusia* to *Hagnagora*.

All species assigned to *Hagnagora* in this revision share distinct wing patterns including a conspicuous white or yellow transversal band or blotch on the forewing. In addition, members of the clades *anicata*, *ephestris* and *mortipax* share a striated pattern on the hindwing underside. The *croceitincta* clade and *Hagnagora subrosea* show remnants of this striation, but the members of the *buckleyi* clade display distinctly different hindwing patterns. Molecular genetic data are available for all groups, but unfortunately with the exception of the *buckleyi* clade and *Hagnagora subrosea*. In a genetic dataset including more than 1,400 species of Ecuadorian geometrid species, the six recorded *Hagnagora* species representing four different clades form a single cluster (Brehm et al. 2013). This strongly suggests that at least these four clades form a monophyletic group (see also Fig. 1). Further molecular genetic data and genitalia dissections are required for members of the *buckleyi* clade to test whether the entire group represents a monophyletic taxon or possibly consists of two distinct lineages.

### Taxonomy of *Hagnagora*

An overview of all taxa is provided in Table 1, and an overview of new type specimens and reference specimens with Bardode Index Numbers (BINs) and GenBank Accessions is provided in Table 2.

**Table 2.** Voucher specimens (types and reference specimens for Barcode Index Numbers (BINs) with identification numbers, GenBank Accession numbers and BINs.

Species	Voucher number	GenBank Accession	Type	BIN
*marionae*	GB 014	AM051082.1	paratype	BOLD:AAE6775
*anicata*	ID 16016	HQ576490	BIN reference	BOLD:AAA7285
*elianne*	07-CRBS-1029	no	**holotype**	BOLD:AAA7283
*unnia*	06-CRBS-0049	no	**holotype**	BOLD:AAA7284
*richardi*	ID 18080	KT208284	**holotype**	BOLD:AAI1950
*richardi*	ID 15855	KT208285	paratype	BOLD:AAI1950
*richardi*	ID 16285	JF859087	paratype	BOLD:AAI1950
*richardi*	BC ZSM Lep 04774	no	paratype	BOLD:AAI1950
*richardi*	ID 17328	GU671808	paratype	BOLD:AAI1950
*richardi*	ID 16119	JF858934	paratype	BOLD:AAI1950
*richardi*	ID 17863	HM380148	paratype	BOLD:AAI1950
*hedwigae*	ID 17397	HM432223	**holotype**	BOLD:AAI1951
*croceitincta*	ID 16293	JF859094	BIN reference	BOLD:AAI6476
*clustimena*	10-SRNP-112230	JF846078	BIN reference	BOLD:AAE6774
*mirandahenrichae*	12-SRNP-103819	no	**holotype**	BOLD:AAF7723
*mirandahenrichae*	07-SRNP-103401	JQ566645	paratype	BOLD:AAF7723
*mirandahenrichae*	07-SRNP-103498	JQ566696	paratype	BOLD:AAF7723
*mirandahenrichae*	11-SRNP-102035	JQ545536	paratype	BOLD:AAF7723
*mirandahenrichae*	11-SRNP-102036	JQ545537	paratype	BOLD:AAF7723
*mirandahenrichae*	12-SRNP-105462	no	paratype	BOLD:AAF7723
*mortipax*	ID 17240	GU671855	BIN reference	BOLD:AAA0650
*discordata*	BC ZSM Lep 44128	no	BIN reference	BOLD:AAA7267
*luteoradiata*	ID 16725	HQ576573	BIN reference	BOLD:AAA7267
*mesenata*	AYK-04-0533-2	KF491827	BIN reference	BOLD:AAH4713
*vittata*	BC LP 0092	no	BIN reference	no BIN

### 1 *buckleyi* clade

#### Hagnagora buckleyi

Taxon classificationAnimaliaLepidopteraGeometridae

Druce, 1885

no assigned BIN

Figs 2 , 3

##### Type locality.

Ecuador, Intaj [possibly Intag, Imbabura province].

##### Remarks.

Druce (1885a) described *Hagnagora buckleyi* and *Hagnagora lex*. The upper- and undersides of the wings in *Hagnagora buckleyi* are very similar, with the colour of the hindwings generally being paler. The forewings feature a deep orange transversal band on a dark brown background, and the hindwings show metallic blue fields between the veins, with three located on the upperside between M_3_ and CuA_2_ and one in the cell, and eight between all veins on the underside. The pattern of the female is similar, with the blue fields extending further on the forewing, including the blotch between veins CuA_2 _and A. In the female, metallic blue scales are also present at the base of the forewing at both the wing upper- and underside.

##### Distribution.

North-western Ecuador.

##### Diagnosis.

The largest species of the clade. The extension of the blue fields is significantly larger than in *Hagnagora lex*. The orange transversal band on the forewing is more saturated and more rounded than in *Hagnagora catagrammina*. Form and extension of the metallic blue blotches are different from those in *Hagnagora catagrammina* (Figs 2–3, 5–6).

#### Hagnagora catagrammina

Taxon classificationAnimaliaLepidopteraGeometridae

Druce, 1885 stat. rev.

no assigned BIN

Figs 5 , 6

##### Type locality.

Nicaragua: Chontales; Panama: Volcán de Chiriqui; Bugaba, 800–1500 ft.

##### Remarks.

Druce (1885b) described *catagrammina* in the same year, but separately from *Hagnagora buckleyi* and *Hagnagora lex*. The taxon was put in synonymy with *buckleyi* by Parsons et al. (1999). As noted by Druce, *catagrammina* is closely related to the other two species of the clade and particularly similar to *Hagnagora buckleyi*. In agreement with Druces’ original description of the three taxa, I revive the species from synonymy with *Hagnagora buckleyi* due to small but overall significant differences of the wing patterns. The morphological differences hint to different species, particularly given the experience from many other species complexes of Neotropical Geometridae in which often more subtle differences – ideally combined with results from genitalia morphology and barcoding – can be observed in different species.

##### Distribution.

Central America, from Nicaragua to Panama.

##### Diagnosis.

The extension of the blue blotches is significantly larger than in *Hagnagora lex*. The transversal band on the forewing is paler and straighter than in *Hagnagora catagrammina*. Form and extension of the metallic blue blotches are different from those in *Hagnagora buckleyi* (Figs 2–3).

#### Hagnagora lex

Taxon classificationAnimaliaLepidopteraGeometridae

Druce, 1885

no assigned BIN

Fig. 4

##### Type locality.

Ecuador (east), [Pastaza], Sarayacu.

##### Remarks.

*Hagnagora lex* was described by Druce (1885a) together with *Hagnagora buckleyi*. While *buckleyi* was collected on the western slopes of the Andes, *Hagnagora lex* originates from the Amazon slopes of the Eastern Andes.

##### Distribution.

Eastern Ecuadorian Andes (Pastaza: Sarayacu).

##### Diagnosis.

Smaller than *Hagnagora buckleyi* and of similar size to *Hagnagora catagrammina*. The extension of the blue blotches is significantly smaller than in *Hagnagora buckleyi*. The form of the transversal band on the forewing is similar to that in *Hagnagora buckleyi*, but the band does not stretch as far towards the wing margins. *Hagnagora lex* is the species with the smallest extensions of metallic blue blotches on the underside, with the upperside completely devoit of these blotches.

### 2 *anicata* clade

#### Hagnagora anicata

Taxon classificationAnimaliaLepidopteraGeometridae

(Felder & Rogenhofer, 1875)

BIN: BOLD:AAA7285

Figs 7 , 8

##### Type locality.

[Colombia], Bogotá.

##### Remarks.

**Figures 7–11.**
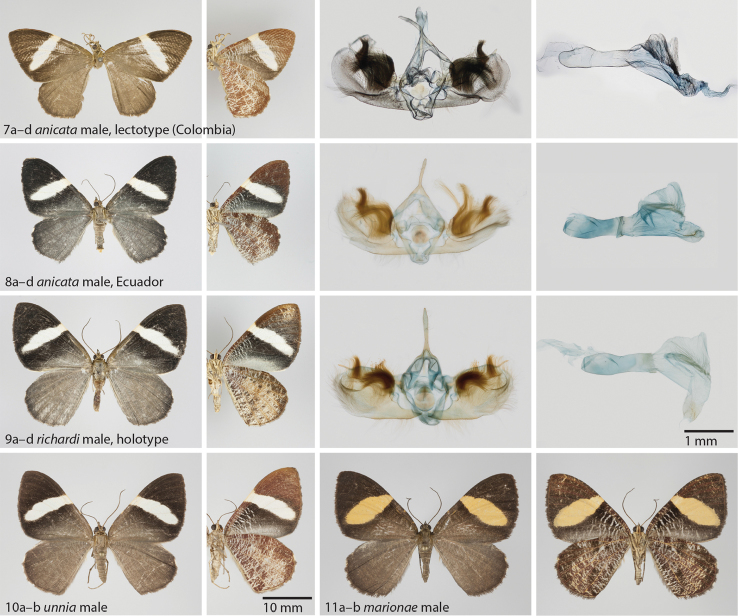
**7** *Hagnagora anicata* (F&R), male lectotype **a** dorsal view **b** ventral view **c** valvae **d** aedeagus **8** *Hagnagora anicata* (F&R), male from Ecuador as reference specimen with Barcode Index Number (BIN) **a** dorsal view **b** ventral view **c** valvae **d** aedeagus **9** *Hagnagora richardi* sp. n., male holotype **a** dorsal view **b** ventral view **c** valvae **d** aedeagus **10** *Hagnagora unnia* Sullivan, male **a** dorsal view **b** ventral view **11** *Hagnagora marionae* Brehm & Sullivan, male **a** dorsal view **b** ventral view.

**Figures 12–14.**
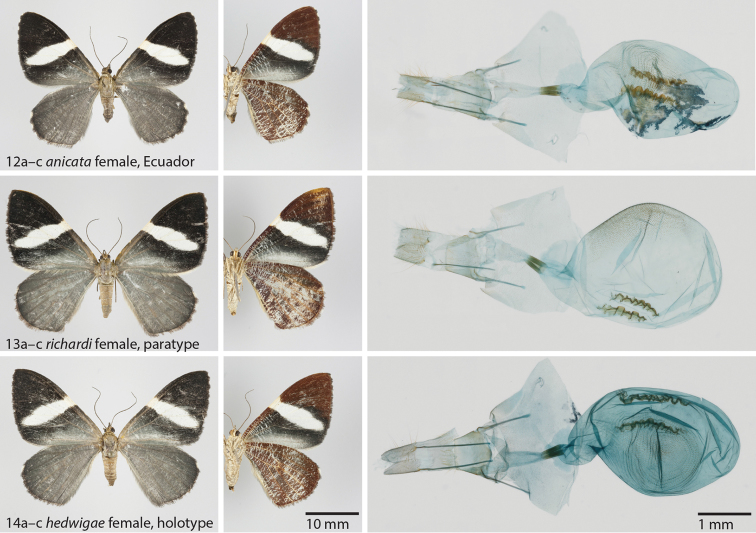
**12** *Hagnagora anicata* (F&R), female from Ecuador as reference specimen with Barcode Index Number (BIN) **a** dorsal view **b** ventral view **c** genitalia **13** *Hagnagora richardi* sp. n., female, paratype **a** dorsal view **b** ventral view **c** genitalia **14** *Hagnagora hedwigae* sp. n., female, paratype **a** dorsal view **b** ventral view **c** genitalia.

*Hagnagora anicata* was re-described with a description also of the male genitalia, by Sullivan (2013). The lectotype is illustrated in Fig. 7. A series of specimens collected in southern Ecuador (1999–2013) (Fig. 8) is indistinguishable from *Hagnagora anicata* and therefore regarded as conspecific. The female (Fig. 12) is larger than the male. A living specimen is shown in Fig. 41 in the typical resting position of these beautiful moths.

##### Distribution.

Apart from its Colombian type locality, *Hagnagora anicata* has recently been collected and barcoded from sites in southern Ecuador to central Bolivia at elevations ranging from 2000 to 2920 m a.s.l.

##### Diagnosis.

Most species of the *Hagnagora anicata* clade are very similar, and the most reliable current method for diagnosis is the COI barcode. *Hagnagora anicata* tends to be smaller than the other species occurring sympatrically, namely *Hagnagora richardi* and *Hagnagora hedwigae*: The wing length of the male (holotype) is only 17.5 mm in comparison to 19 mm in the male holotype of *Hagnagora richardi*. The structures of the female signum are also more complex than in *Hagnagora richardi*, but similar to those in *Hagnagora hedwigae*. The uncus of the male is smaller and shorter than in *Hagnagora richardi*. Aedeagi of the known males are (*Hagnagora anicata* and *Hagnagora richardi*) similar. COI barcode: The minimum observed distance to the presumbably most closely related species (*Hagnagora hedwigae*) is 3.1%.

#### Hagnagora elianne

Taxon classificationAnimaliaLepidopteraGeometridae

Sullivan, 2011

Not figured (very similar to H. unnia)

BIN (paratype): BOLD:AAA7283

Voucher 07-CRBS-1029

##### Type locality (holotype).

Costa Rica: Alajuela Province, Poás Volcano National Park, 2500 m.

##### Remarks.

*Hagnagora elianne* was described and illustrated by Sullivan (2011). The species closely resembles the other species in the *Hagnagora anicata* clade, particularly *Hagnagora unnia*.

##### Distribution.

The species has recently been collected and barcoded in Honduras (Cortes Province) and in several provinces of Costa Rica at elevations ranging from 1480 to 2840 m a.s.l.

##### Diagnosis.

Males are on average slightly larger than males in *Hagnagora unnia* and can be distinguished from *Hagnagora anicata* by a swollen as opposed to a gently tapered distal half of the uncus and by the absence of a moderately large, upcurved spine at the end of the costa in *Hagnagora elianne* (Sullivan 2011). Females may be distinguished from females of *Hagnagora unnia* by their longer, more complex signum. COI barcode: The minimum observed distance to the presumably most closely related species (*Hagnagora unnia*) is 5.0%.

#### Hagnagora unnia

Taxon classificationAnimaliaLepidopteraGeometridae

Sullivan, 2011

BIN (paratype): BOLD:AAA7284

Voucher 06-CRBS-0049

Fig. 10

##### Type locality

(holotype): Costa Rica, Cartago Province, Tapantí National Park, 1275 m.

##### Remarks.

*Hagnagora unnia* was recently described and illustrated by Sullivan (2011).

##### Distribution.

The species is known from several provinces in Costa Rica at elevations ranging from 587 to 2840 m a.s.l.

##### Diagnosis.

The species closely resembles other species of the *Hagnagora anicata* clade, particularly *Hagnagora elianne*; see there for a diagnosis. COI barcode: The minimum observed distance to the presumably most closely related species (*Hagnagora elianne*) is 5.0%.

#### Hagnagora marionae

Taxon classificationAnimaliaLepidopteraGeometridae

Brehm & Sullivan, 2005

BIN (paratype): BOLD:AAE6775

GenBank Accession: AM051082

Fig. 11

##### Type locality.

Costa Rica, Heredia province, Braulio Carrillo National Park, Volcán Barva, 2730 m a.s.l.

##### Remarks.

*Hagnagora marionae* was described and illustrated by Brehm and Sullivan (2005).

##### Distribution.

The species has been collected only at two high mountain areas in Costa Rica at elevations > 2500 m a.s.l.

##### Diagnosis.

The species resembles the other species of the *Hagnagora anicata* clade, but is easily distinguished by large orange-yellow blotches on the forewing. Males have a spatula-shaped uncus. COI barcode: The minimum observed distance to the presumably most closely related species (*Hagnagora richardi*) is 6.6%.

#### Hagnagora richardi

Taxon classificationAnimaliaLepidopteraGeometridae

Brehm sp. n.

http://zoobank.org/406E12C4-4231-49F2-BE51-61E504E395F7

BIN (holotype): BOLD:AAI1950

Voucher ID 18080

GenBank Accession: KT208284

Figs 9 , 13

##### Type material.

**Holotype**: male (Fig. 9): Ecuador, Loja province, Parque Nacional Podocarpus, Cajanuma, 04°06.85'S, 79°10.47'W, 2916 m, 20 November 2008, G. Brehm leg. (ID 18080, genitalia preparation, barcode sequence 658 bp) (PMJ).

**Paratypes**: (deposited in CISEC, PMJ, RCGB, ZSM) 4 males, 2 females. 1 female: same as holotype but 04°06.86'S, 79°10.46'W, 2897 m, F. Bodner leg. (ID 15855, barcode sequence 658 bp); 1 female (Fig. 13): Ecuador, Zamora Chinchipe, Reserva Biológica San Francisco, 03°58.72'S, 79°04.44'W, 2180 m, 16 November 2008, F. Bodner leg. (ID 16285, barcode sequence 658 bp); 1 male same as previous but 28 October 1999, D. Süßenbach leg. (BC ZSM Lep 04774, barcode sequence 529 bp); 1 male: same as previous but 03°59.65'S, 79°04.10'W, 2670 m, G. Brehm leg. (ID 17328, barcode sequence 658 bp); 1 male as previous but 03°59.68'S, 79°04.10'W, 2677 m, 18 November 2008 (ID 16119, barcode sequence 658 bp); 1 male as previous but 25 November 2008 (ID 17863, barcode sequence 621 bp).

##### Description.

As illustrated in Figs 9, 13. The wing length of the holotype (male) is 19 mm. The wing length of a female paratype (Fig. 13) is 21 mm.

##### Distribution.

Only known from a small region around Podocarpus National Park, provinces Zamora-Chinchipe and Loja, Ecuador, with an observed elevational range of 2180–3021 m a.s.l. Apart from the

##### Type locality

and nearby sites, specimens were collected at elevations at ca. 3000 m at Cerro Toledo in the same National Park (04°23'S, 79°07'W). However, this record is not fully reliable because genitalia preparation or barcoding was not conducted for these specimens.

##### Diagnosis.

Closely resembles other species of the *Hagnagora anicata* clade. On average significantly larger than *Hagnagora anicata*, but the female has about the same size as *Hagnagora hedwigae*. The uncus of the male is larger and broader than in *Hagnagora anicata*. The signum of the bursa copulatrix is less complex than in *Hagnagora anicata* and *Hagnagora hedwigae*. Easily distinguishable from *Hagnagora marionae* by the cream-white colour of the blotches on the forewing. COI barcode: The minimum observed distance to the presumably most closely related species (*Hagnagora marionae*) is 6.6%.

##### Etymology.

*Hagnagora richardi* is named in honour of Richard Philipp from Jena, Germany, in recognition of his and his parents’ support for the taxonomy of Neotropical geometrid moths.

#### Hagnagora hedwigae

Taxon classificationAnimaliaLepidopteraGeometridae

Brehm sp. n.

http://zoobank.org/91A46B5D-DF10-42A8-97B2-46E0D5D7E086

BIN (holotype): BOLD:AAI1951

Voucher ID 17397

GenBank Accession HM432223

Fig. 14

##### Type material.

**Holotype**: female (Fig. 14): Ecuador, Loja province, Reserva Biológica San Francisco, 03°59.68'S, 79°04.10'W, 2677 m, 25 November 2008, G. Brehm leg. (ID 17397, genitalia preparation, barcode sequence 595 bp) (PMJ).

##### Description.

As illustrated in Fig. 14.

##### Distribution.

Only a single female is known from *Hagnagora hedwigae* collected in southern Ecuador (2677 m). The wing length of the holotype (female) is 21 mm (same size as *richardi*).

##### Diagnosis.

Resembles most closely *Hagnagora anicata* and *Hagnagora richardi*, but is larger than *Hagnagora anicata*, and the signum of the bursa copulatrix is more complex than in *Hagnagora richardi*. COI barcode: The minimum observed distance to the presumably most closely related species (*Hagnagora anicata*) is 3.1%.

##### Etymology.

*Hagnagora hedwigae* is named in memory of Hedwig Seppelt (*1919 in Baumgarten, Silesia; † 2013 in Korschenbroich, Germany). Mrs Seppelt loved nature, and she took care that birds, small animals and insects found a habitat in her garden. The name is given in recognition of support for the taxonomy of Neotropical geometrid moths provided by her daughter-in-law Irmgard and her son Winfried Seppelt.

### 3 *croceitincta* clade

#### Hagnagora croceitincta

Taxon classificationAnimaliaLepidopteraGeometridae

(Dognin, 1892)

BIN: BOLD:AAI6476

Figs 15–17

epimena (Bastelberger, 1908): Type locality. Peru (east), Cuschi [Cushi]

##### Type locality.

[Ecador, (south)], Loja surroundings.

##### Remarks.

**Figures 15–22.**
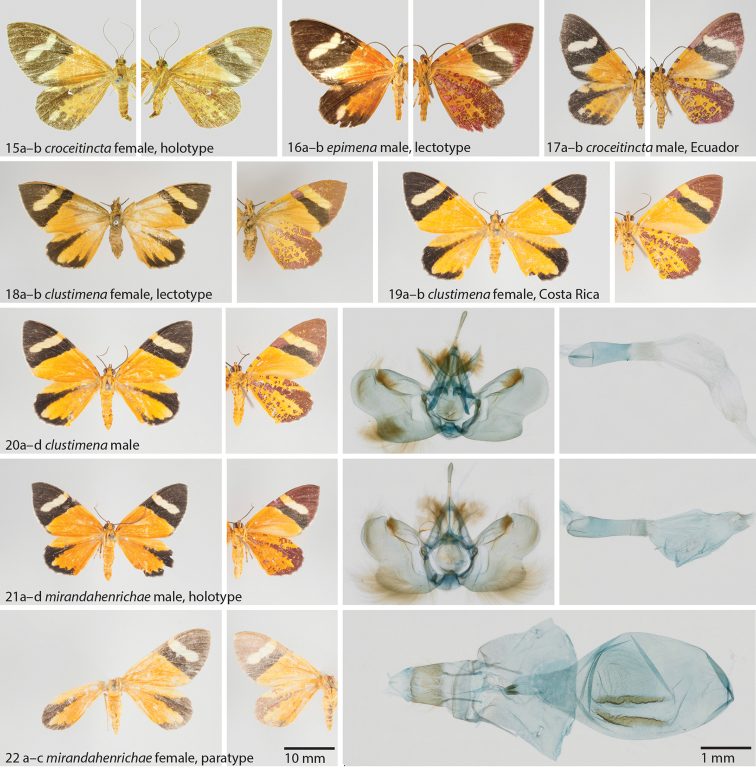
**15** *Hagnagora croceitincta* (Dognin) female, holotype **a** dorsal view **b** ventral view **16** *Hagnagora epimena* (Bastelberger) male, lectotype **a** dorsal view **b** ventral view **17** *Hagnagora croceitincta* male from Ecuador as reference specimen with Barcode Index Number (BIN) **a** dorsal view **b** ventral view **18** *Hagnagora clustimena* (Druce) female, lectotype **a** dorsal view **b** ventral view **19** *Hagnagora clustimena* female from Costa Rica as reference specimen with Barcode Index Number (BIN) **20** *Hagnagora clustimena* male from Costa Rica as reference specimen with Barcode Index Number (BIN) **a** dorsal view **b** ventral view **c** valvae **d** aedeagus **21** *Hagnagora mirandahenrichae* Brehm sp. n. male, holotype **a** dorsal view **b** ventral view **c** valvae **d** aedeagus **22** *Hagnagora mirandahenrichae* female, paratype **a** dorsal view **b** ventral view **c** genitalia.

*Hagnagora croceitincta* was described by Dognin from southern Ecuador where it has recently been collected in montane forests (Brehm 2002). As one of the largest known *Hagnagora* species, it is conspicuously coloured, with orange, dark brown and white patterns. The taxon *epimena* (Bastelberger) remains in synonymy because the lectotype specimen (Fig. 16) does not show any particular differences to the type specimen of *Hagnagora croceitincta* (Fig. 15).

##### Distribution.

Recently collected and barcoded specimens were sampled from central Colombia to southeastern Peru at elevations between 1750 and 2540 m a.s.l.

##### Diagnosis.

On average larger than the closely related species *Hagnagora clustimena* and *Hagnagora mirandahenrichae*: Forewing length of the female holotype reaches 23 mm in comparison to about 20 mm in the other species. On the forewing, the white transversal blotch does not stretch to the costal margin as seen in the other two species, and the apical, dark-brown area reaches beyond veins 1A+2A. The species is also generally more vividly coloured than the other species in this clade, with white spots on the forewing between veins CuA_2_ and 1A+2A and around M_3_ on the upperside of the hindwing. COI barcode: The minimum observed distance to the presumably most closely related species (*Hagnagora mirandahenrichae*) is 7.1%.

#### Hagnagora clustimena

Taxon classificationAnimaliaLepidopteraGeometridae

(Druce, 1893)

BIN: BOLD:AAE6774

Figs 18–20

##### Type locality.

Mexico: Coatepec.

##### Remarks.

*Hagnagora clustimena* was originally assigned by Druce to *Heterusia* and then transferred to *Hagnagora* by Parsons et al. (1999). *Hagnagora clustimena* and *Hagnagora croceitincta* appear to occur allopatrically.

##### Distribution.

Besides the type specimens described by Druce from Mexico and Panama, recently collected and barcoded specimens were sampled in Honduras and Costa Rica between 850 and 1550 m a.s.l.

##### Diagnosis.

On average smaller than *Hagnagora croceitincta* and slightly larger than *Hagnagora mirandahenrichae* (see *Hagnagora croceitincta*). The white transversal blotch on the forewing stretches to the costal margin and the apical dark-brown area reaches vein CuA_2_, as also observed in *mirandahenrichae*. *Hagnagora clustimena* is slightly paler than *Hagnagora mirandahenrichae*. The male genitalia of both species are similar, but the valves are broader and differently shaped to *mirandahenrichae*. COI barcode: The minimum observed distance to the presumably most closely related species (*Hagnagora mirandahenrichae*) is 4.6%.

#### Hagnagora mirandahenrichae

Taxon classificationAnimaliaLepidopteraGeometridae

Brehm sp. n.

http://zoobank.org/4C4FF729-5872-412A-BEF4-2428DA894237

BIN (holotype) BOLD:AAF7723

Figs 21–22

##### Type locality.

Costa Rica, Área de Conservación Guanacaste, Guanacaste province.

##### Type material.

**Holotype**: male (Fig. 21): Costa Rica, Guanacaste province, Área de Conservación Guanacaste, Sector Santa Maria, Mirador Santa Maria, 10.766° N, 85.301°W, 920 m a.s.l., 20 June 2012, S. Rios & R. Franco leg. (voucher 12-SRNP-103819, genitalia preparation, barcode sequence 658 bp) (PMJ).

**Paratypes**: (deposited in PMJ, USNM) 5 males, 1 female. Costa Rica, Guanacaste province, Área de Conservación Guanacaste, Sector Pitilla, Estacion Pitilla, 10.989° N, 85.426° W, 675 m a.s.l.; 1 female (Fig. 22) 16 May 2007, F. Quesada & R. Franco leg. (voucher 07-SRNP-103401, genitalia preparation, barcode sequence 658 bp), 1 male same as previous but 17 May 2007 (voucher 07-SRNP-103498), 2 males, 02 Apr 2011, H. Cambronero & S. Rios leg. (vouchers 11-SRNP-102035 and 11-SRNP-102036, barcode sequences 658 bp), 1 male 12 November 2012, R. Franco & H. Cambronero leg. (voucher 12-SRNP-105462).

##### Description.

As illustrated in Figs 21, 22.

##### Distribution.

Only known from sectors Santa Maria and Pitilla from Área de Conservación Guanacaste, province Guanacaste, NW Costa Rica, at elevations ranging from 675–920 m a.s.l., and therefore with a lower elevational range than *Hagnagora clustimena* (observed: 850–1550 m a.s.l.).

##### Diagnosis.

Easily distinguished from *Hagnagora croceitincta* by its wing patterns (see diagnosis in that species). The yellow ground colour of *Hagnagora mirandahenrichae* is slightly more intensive than in *Hagnagora clustimena*. The male genitalia of both species are similar, but the valves of *mirandahenrichae* are narrower and have a different shape to *clustimena*. COI barcode: The minimum observed distance to the presumably closest relative, *Hagnagora clustimena*, is 4.6%.

##### Etymology.

*Hagnagora mirandahenrichae* is named in honour of Ms. Miranda Henrich of California in recognition of her and her mother’s critical support for understanding the taxonomy and biodiversity development of the Área de Conservación Guanacaste (ACG) in northwestern Costa Rica, where this species has been found by the ACG caterpillar inventory (Janzen et al. 2014).

### 4 *mortipax* clade

#### Hagnagora mortipax

Taxon classificationAnimaliaLepidopteraGeometridae

(Butler, 1872)

BIN: BOLD:AAA0650

Figs 23–25

flavipectus (Warren, 1897): Type locality. [Colombia], Bogotá.

##### Type locality.

Costa Rica.

##### Remarks.

*Hagnagora mortipax* is one of the earliest described species in the genus and among the smallest *Hagnagora* species. Together with *Hagnagora luteoradiata* it also has the largest known geographical range. The taxon *flavipectus* remains in synonymy because it falls within the confirmed geographical range of *mortipax* and shows no significant deviations from the type specimen of *mortipax*. In comparison to the type specimen, the extension of the large white blotch on the forewing is smaller in Ecuadorian specimens, where it does not reach the costal margin. Since the barcode sequences of Costa Rican and Ecuadorian populations are nearly identical, all respective specimens are treated as members of the same species, and slight differences in wing patterns are regarded as geographical variability.

##### Distribution.

Recently sampled and barcoded material is either from Costa Rica (Fig. 24) or Ecuador (Fig. 25), from elevations ranging from 540–2180 m a.s.l., and additional material from Ecuador falls within the same elevational range (Brehm 2002).

**Figures 23–29.**
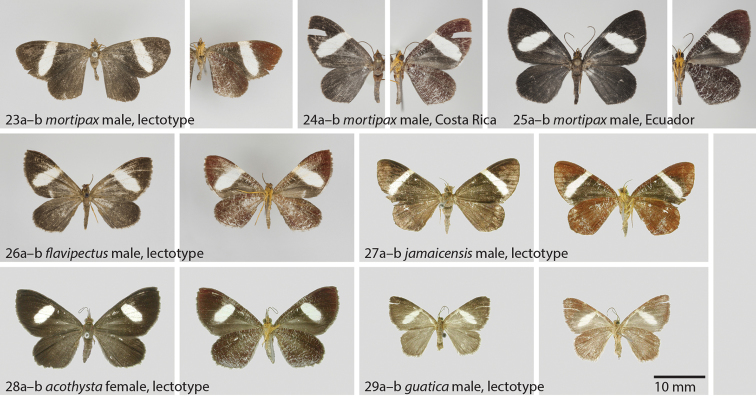
**23** *Hagnagora moripax* (Druce) male, lectotype **a** dorsal view **b** ventral view **24** *Hagnagora mortipax* male from Costa Rica as reference specimen with Barcode Index Number (BIN) **a** dorsal view **b** ventral view **25** *Hagnagora mortipax* male from Ecuador as reference specimen with Barcode Index Number (BIN) **a** dorsal view **b** ventral view **26** *Hagnagora flavipectus* (Warren) male, holotype **a** dorsal view **b** ventral view **27** *Hagnagora mortipax jamaicensis* (Schaus) male, lectotype **a** dorsal view **b** ventral view (photo USNM) **28** *Hagnagora mortipax acothysta* (Schaus) female, lectotype **a** dorsal view **b** ventral view (photo USNM) **29** *Hagnagora guatica* (Schaus) female, lectotype **a** dorsal view **b** ventral view (photo USNM).

##### Diagnosis.

The upper side of the wing in *Hagnagora mortipax* has a dark brown base colour with a large cream-white blotch on the forewing. This blotch almost reaches the outer margin, also either reaching the costal margin (Costa Rican specimens), or scantily not (Ecuadorian specimens). The white blotch is narrower in *Hagnagora jamaicensis* (Fig. 27), and significantly smaller, and separated from the outer margin, in *Hagnagora acothysta* from Brazil. All three species are significantly larger than *Hagnagora guatica*.

#### Hagnagora jamaicensis

Taxon classificationAnimaliaLepidopteraGeometridae

(Schaus, 1901) stat. rev.

no assigned BIN

Fig. 27

##### Type locality.

Jamaica.

##### Remarks.

Originally described as a *Heterusia* species by Schaus (1901), this taxon was down-ranked as a subspecies of *mortipax* by Parsons et al. (1999). In my view, the significantly different wing pattern in *jamaicensis* justifies Schaus’ original species rank, but further evidence from barcoding is desirable in order to consolidate its species status.

##### Distribution.

Jamaica.

##### Diagnosis.

In contrast to the other taxa in the *mortipax* clade, this species displays a very narrow, cream-white transversal band on the forewings. The striation on the underside of the hindwing is reduced in comparison to *mortipax* and *acothysta*.

#### Hagnagora acothysta

Taxon classificationAnimaliaLepidopteraGeometridae

(Schaus, 1901) stat. rev.

no assigned BIN

Fig. 28

##### Type locality.

[Brazil], Parana, Castro.

##### Remarks.

Together with *jamaicensis*, Schaus (1901) originally placed this species in the genus *Heterusia*. It was then ranked down as a subspecies of *mortipax* by Parsons et al. (1999). The major characteristic of *acothysta* is the reduction of the white transversal band (found both in *mortipax* and *jamaicensis*) to a smaller blotch that reaches about half the area found in *mortipax*. As in *jamaicensis*, further evidence from barcoding is desirable for the consolidation of the species status.

##### Distribution.

Brazil.

##### Diagnosis.

Unlike *mortipax* and *jamaicensis*, this species shows no white transversal band on the forewing, but rather a reduced blotch that reaches only about 50% of the size observed in *mortipax*.

#### Hagnagora guatica

Taxon classificationAnimaliaLepidopteraGeometridae

(Schaus, 1927)

no assigned BIN

Fig. 29

##### Type locality.

Guatemala, [Quetzaltenango Department], Volcán Sta. Maria.

##### Remarks.

Schaus described *guatica* as belonging to *Scordylia* Gn (a junior synonym of *Heterusia*). The wing pattern of *guatica* strongly resembles that of other members in the *mortipax* clade, but the species lacks the typical striation on the underside of the hindwing. Further evidence from barcoding and the study of the genitalia will help to better understand the relationships of this species with other species of the *mortipax* clade.

##### Distribution.

Guatemala.

##### Diagnosis.

By far the smallest *Hagnagora* species. The species lacks the typical striation on the underside of the hindwing found in all other members of the *mortipax* clade.

### 5 *ephestris* clade

#### Hagnagora ephestris

Taxon classificationAnimaliaLepidopteraGeometridae

(Felder & Rogenhofer, 1875)

no assigned BIN

Fig. 30

##### Type locality.

[Colombia], Bogota.

##### Remarks.

Felder & Rogenhofer described this species from Colombia. It closely resembles *Hagnagora discordata* and *Hagnagora luteoradiata*. Parsons et al. (1999) put *luteoradiata* in synonymy with *ephestris*, but freshly collected material from Costa Rica and Ecuador shows that *luteoradiata* consistently lacks yellow blotches on the hindwing. It appears therefore to be more likely that *ephestris* is a junior synonym of *discordata*, and an increased knowledge of COI sequences could help to solve this question. Given the current state of knowledge, it appears to be the most appropriate solution to revive *luteoradiata* from synonymy and to treat the other two taxa as full species.

**Figures 30–35.**
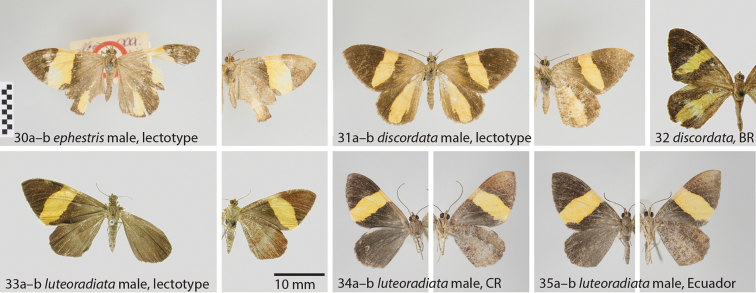
**30** *Hagnagora ephestris* (F&R) male, lectotype **a** dorsal view **b** ventral view **31** *Hagnagora discordata* male, lectotype **a** dorsal view **b** ventral view **32** *Hagnagora discordata* male (ZSM Lep 44128) from Brazil as reference specimen with Barcode Index Number (BIN) (photo ZSM) **33** *Hagnagora luteoradiata* (T-M) male, lectotype **a** dorsal view **b** ventral view **34** *Hagnagora luteoradiata* (T-M) male from Costa Rica (CR) as reference specimen with Barcode Index Number (BIN) **a** dorsal view **b** ventral view **35** *Hagnagora luteoradiata* (T-M) male from Ecuador as reference specimen with Barcode Index Number (BIN) **a** dorsal view **b** ventral view.

##### Distribution.

Colombia.

##### Diagnosis.

Both *ephestris* and *discordata* show a pronounced yellow blotch on the hindwings that is absent in *luteoradiata*. Different from *discordata*, the yellow transversal band on the forewing of *Hagnagora ephestris* reaches the outer margin of the wing. Moreover, the band is broader than in *discordata*, whereas the yellow field of the hindwing is narrower, particularly in the proximate half of the wing.

#### Hagnagora discordata

Taxon classificationAnimaliaLepidopteraGeometridae

(Guenée [1858])

BIN: BOLD:AAA7267 (together with luteoradiata)

Figs 31 , 32

##### Type locality.

[Chile], Valparaíso [possibly incorrect locality].

##### Remarks.

The oldest described *Hagnagora* species, assigned by Guenée to *Scordylia* Gn (= *Heterusia*).

##### Type locality

Given as Valparaíso, [Chile], requires confirmation. The cool-dry climate of this Chilean lowland region differs strongly from the wet montane habitats where other *Hagnagora* species are typically found.

##### Distribution.

Apart from the doubtful type locality in Chile, recently collected specimens were sampled in Santa Catarina, Brazil (27°S), at elevations of 1300 m a.s.l.

##### Diagnosis.

Both *discordata* and *ephestris* show a pronounced yellow blotch on the hindwings that is absent in *luteoradiata*. The yellow transversal band on the forewing is narrower than in *ephestris*, and it does not reach the outer margin of the wing. The yellow blotch on the hindwing is much broader than in *Hagnagora discordata*. COI barcode: The minimum observed distance of Brazilian *Hagnagora discordata* is 2.3% to *Hagnagora luteoradiata* from Costa Rica and 2.6% to *Hagnagora luteoradiata* from Ecuador. These short distances suggest a relatively young split within this species clade.

#### Hagnagora luteoradiata

Taxon classificationAnimaliaLepidopteraGeometridae

(Thierry-Mieg, 1892) stat. rev.

BIN: BOLD:AAA7267 (together with discordata)

Figs 33–35

##### Type locality.

Costa Rica.

##### Remarks.

*Hagnagora luteoradiata* was put in synonymy with *Hagnagora ephestris* by Parsons et al. (1999). However, *luteoradiata* specimens consistently do not show any yellow blotches on the hindwing as observed in *ephestris* and *clustimena*. Barcoded specimens from Costa Rica (Fig. 34) and Ecuador (Fig. 35) are genetically very similar (distance only ca. 1.1%) and, together with the highly similar appearance, are therefore regarded as conspecific. The *ephestris* type specimen from Bogotá, Colombia, falls within the geographical range of the *luteoradiata* specimens, but shows a different wing pattern, i.e. a prominent yellow blotch on the hindwing and a different shape of the blotch of the forewing. The taxon *luteoradiata* is therefore revived from synonymy.

##### Distribution.

Costa Rica to Ecuador. Observed elevational range in Ecuador 1800–2890 m and 560–1480 m in Costa Rica.

##### Diagnosis.

The most prominent difference is the absence of any yellow blotches on the hindwing that are present both in *ephestris* and *discordata*. The transversal yellow band on the forewing is broader than in *discordata*, and has a different shape than in *ephestris*.

### 6 *subrosea*

#### Hagnagora subrosea

Taxon classificationAnimaliaLepidopteraGeometridae

(Warren, 1909)

no assigned BIN

Fig. 36

##### Type locality.

Peru (south east), Carabaya, Oconeque, 7000 ft.

##### Remarks.

Warren originally assigned *subrosea* to *Cophocerotis* Warren, but the genus-defining type species, *Cophocerotis jaspeata* (Dognin), does not show the two prominent white transversal bands of the forewing present in *subrosea*. Parsons et al. (1999) transferred the species to *Hagnagora*. Barcoding and genitalia dissections of fresh specimens from this species are required, but judging from the two transversal bands, *subrosea* might indeed be associated with the *croceitincta* clade.

##### Distribution.

Peru.

##### Diagnosis.

*Hagnagora subrosea* has a unique combination of a pale brown wing colour with two white transversal bands on the forewings not found in any other species of *Hagnagora*.

### Species excluded from *Hagnagora*

The following species are provisionally removed from the genus *Hagnagora* and set in quotation marks, following the convention applied by Parsons et al. (1999). *“Hagnagora” ignipennis* (Fig. 37) from Colombia lacks most of the characteristics typical for *Hagnagora*, notably transversal bands on the forewing. *“Hagnagora” mesenata*, *“Hagnagora” vittata* and *“Hagnagora” ceraria* (Figs 38–40) appear to be closely related to each other, but the wing pattern and particularly the wing shape diverge strongly from other species treated as “true” *Hagnagora* in this paper. A full barcode sequence is available for *“Hagnagora” mesenata*, and a 380 bp fragment of the COI gene is available for *“Hagnagora” vittata*. Both sequences reveal that these species are probably not congeneric with “true” *Hagnagora*. It is possible that the clades around *ignipennis* and *vittata* represent undescribed Larentiinae genera, and both cases require closer examination and a thorough revision of Neotropical Larentiinae.

**Figures 36–40.**
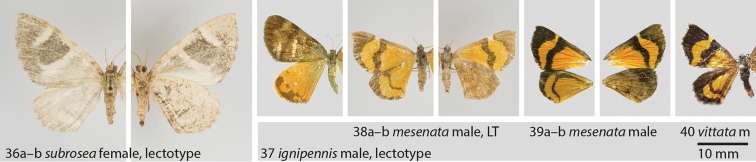
**36** *Hagnagora subrosea* (Warren) female, lectotype **a** dorsal view **b** ventral view **37** *“Hagnagora” ignipennis* (Dognin) male, lectotype dorsal view **38** *“Hagnagora” mesenata* (F&R) male, lectotype (LT) **a** dorsal view **b** ventral view **39** *“Hagnagora” mesenata* male (AYK-04-0533-2) from Chile as reference specimen with Barcode Index Number (BIN) **a** dorsal view **b** ventral view (photo K Mitter) **40** *“Hagnagora” vittata* (Philippi) male (m) (BC LP 0092) from Chile as reference specimen with 380 bp COI fragment, dorsal view (photo LE Parra).

**Figures 41–43.**
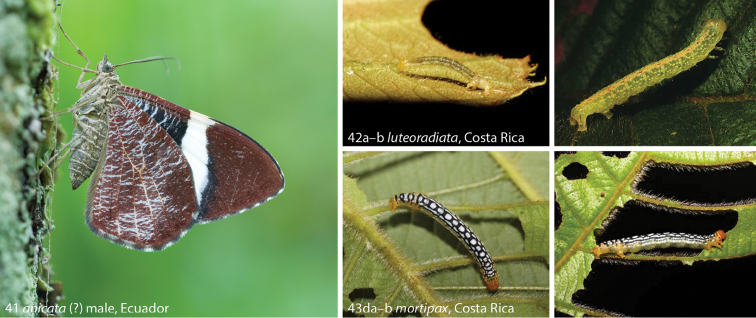
*Hagnagora* living specimens **41** *Hagnagora anicata* (?), Ecuador, Zamora Chinchipe, Estación Biológica San Francisco, 22 November 2008 in typical resting habitus, but alert because of disturbance by the photographer. The tympanal organ at the base of the abdomen is well visible **42** *Hagnagora luteoradiata* from Costa Rica **a** young caterpillar (09-SRNP-31840-DHJ458869) **b** caterpillar in last instar (09-SRNP-31840-DHJ458860) **43** *Hagnagora mortipax* caterpillar from Costa Rica **a** dorsal view (14-SRNP-3240-DHJ487561) **b** lateral view (14-SRNP-3240-DHJ487557).

#### “Hagnagora” ignipennis

Taxon classificationAnimaliaLepidopteraGeometridae

(Dognin, 1913)

no assigned BIN

Fig. 37

##### Type locality.

Colombia, Bogotá, 2800–3200 m.

#### “Hagnagora” mesenata

Taxon classificationAnimaliaLepidopteraGeometridae

(Felder & Rogenhofer, 1875)

assigned BIN: BOLD:AAH4713 of voucher specimen AYK-04-0533-2 from Chile

GenBank Accession: KF491827

Figs 38 , 39

##### Type locality.

Chile.

#### “Hagnagora” vittata

Taxon classificationAnimaliaLepidopteraGeometridae

(Philippi, 1859)

no BIN assigned but 380 bp COI fragment of voucher specimen BC LP 0092 from Chile

Fig. 40

ceraria (Molina, 1782): Type locality. Chile

##### Type locality.

Chile, Provincia de Valdivia.

## Supplementary Material

XML Treatment for Hagnagora buckleyi

XML Treatment for Hagnagora catagrammina

XML Treatment for Hagnagora lex

XML Treatment for Hagnagora anicata

XML Treatment for Hagnagora elianne

XML Treatment for Hagnagora unnia

XML Treatment for Hagnagora marionae

XML Treatment for Hagnagora richardi

XML Treatment for Hagnagora hedwigae

XML Treatment for Hagnagora croceitincta

XML Treatment for Hagnagora clustimena

XML Treatment for Hagnagora mirandahenrichae

XML Treatment for Hagnagora mortipax

XML Treatment for Hagnagora jamaicensis

XML Treatment for Hagnagora acothysta

XML Treatment for Hagnagora guatica

XML Treatment for Hagnagora ephestris

XML Treatment for Hagnagora discordata

XML Treatment for Hagnagora luteoradiata

XML Treatment for Hagnagora subrosea

XML Treatment for “Hagnagora” ignipennis

XML Treatment for “Hagnagora” mesenata

XML Treatment for “Hagnagora” vittata
